# Event-free survival after ^68^ Ga-PSMA-11 PET/CT in recurrent hormone-sensitive prostate cancer (HSPC) patients eligible for salvage therapy

**DOI:** 10.1007/s00259-022-05741-9

**Published:** 2022-02-26

**Authors:** Francesco Ceci, Guido Rovera, Giuseppe Carlo Iorio, Alessia Guarneri, Valeria Chiofalo, Roberto Passera, Marco Oderda, Sara Dall’Armellina, Virginia Liberini, Serena Grimaldi, Marilena Bellò, Paolo Gontero, Umberto Ricardi, Désirée Deandreis

**Affiliations:** 1 Nuclear Medicine, Department of Medical Sciences, AOU Città Della Salute E Della Scienza Di Torino, University of Turin, Turin, Italy; 2 Division of Nuclear Medicine, IEO European Institute of Oncology IRCCS, Milan, Italy; 3 Department of Oncology and Hemato-Oncolology, University of Milan, Milan, Italy; 4 Nuclear Medicine Department, S. Croce E Carle Hospital, Cuneo, Italy; 5 Section of Nuclear Medicine, University Department of Radiological Sciences and Haematology, Università Cattolica del Sacro Cuore, Rome, Italy; 6 Radiation Oncology, Department of Oncology, AOU Città Della Salute E Della Scienza Di Torino, Turin, Italy; 7 Urology, Department of Surgical Sciences, AOU Città Della Salute E Della Scienza Di Torino, University of Turin, Turin, Italy; 8 Department of Oncology, University of Turin, Turin, Italy

**Keywords:** PSMA PET, Event-free survival, Prostate cancer, Hormone-sensitive prostate cancer, Prostate cancer survival

## Abstract

**Background/aim:**

Prostate-specific-membrane-antigen/positron emission tomography (PSMA-PET) detects with high accuracy disease-recurrence, leading to changes in the management of biochemically-recurrent (BCR) prostate cancer (PCa). However, data regarding the oncological outcomes of patients who performed PSMA-PET are needed. The aim of this study was to evaluate the incidence of clinically relevant events during follow-up in patients who performed PSMA-PET for BCR after radical treatment.

**Materials and methods:**

This analysis included consecutive, hormone-sensitive, hormone-free, recurrent PCa patients (HSPC) enrolled through a prospective study. All patients were eligible for salvage therapy, having at least 24 months of follow-up after PSMA-PET. The primary endpoint was the Event-Free Survival (EFS), defined as the time between the PSMA-PET and the date of event/last follow-up. The Kaplan–Meier method was used to estimate the EFS curves. EFS was also investigated by Cox proportional hazards regression. Events were defined as death, radiological progression, or PSA recurrence after therapy.

**Results:**

One-hundred and seventy-six (*n* = 176) patients were analyzed (median PSA 0.62 [IQR: 0.43–1.00] ng/mL; median follow-up of 35.4 [IQR: 26.5–40.3] months). The EFS was 78.8% at 1 year, 65.2% (2 years), and 52.2% (3 years). Patients experiencing events during study follow-up had a significantly higher median PSA (0.81 [IQR: 0.53–1.28] vs 0.51 [IQR: 0.36–0.80] ng/mL) and a lower PSA doubling time (PSAdt) (5.4 [IQR: 3.7–11.6] vs 12.7 [IQR: 6.6–24.3] months) (*p* < 0.001) compared to event-free patients. The Kaplan–Meier curves showed that PSA > 0.5 ng/mL, PSAdt ≤ 6 months, and a positive PSMA-PET result were associated with a higher event rate (*p* < 0.01). No significant differences of event rates were observed in patients who received changes in therapy management after PSMA-PET vs. patients who did not receive therapy changes. Finally, PSA > 0.5 ng/mL and PSAdt ≤ 6 months were statistically significant event-predictors in multivariate model (*p* < 0.001).

**Conclusion:**

Low PSA and long PSAdt were significant predictors of longer EFS. A lower incidence of events was observed in patients having negative PSMA-PET, since longer EFS was significantly more probable in case of a negative scan.

**Supplementary Information:**

The online version contains supplementary material available at 10.1007/s00259-022-05741-9.

## Introduction

The clinical management of prostate cancer (PCa) patients affected by biochemical recurrence (BCR) after radical therapy (either surgery or radiotherapy) has been recently influenced by the introduction of new generation imaging [1]. Prostate specific membrane antigen/positron emission tomography (PSMA-PET) emerged as one of the leading diagnostic procedure to investigate PCa, showing superior diagnostic accuracy compared to other molecular imaging techniques (including choline-PET [2] and fluciclovine-PET [3]), to correctly locate the site of the recurrence [4, 5]. PSMA-PET can provide accurate disease staging and significantly influence the management of recurrent PCa, leading to more effective imaging-guided approaches thanks to an improved target delineation [4, 6]. According to the literature, introducing PSMA-PET in the management of recurrent PCa generates a change in therapy management in nearly 50% of patients [6–8]. At present, data derived from phase III trials enrolling large cohorts of patients and designed to assess the efficacy of PSMA-guided radiotherapy are still awaited [9]. However, preliminary results support the use of new generation imaging to guide salvage radiotherapy (SRT) or stereotactic ablative radiotherapy (SABR) [9–11]. Biochemical responses from PET-based SRT appears to be statistically superior to the response in patients who undergo conventional imaging-based radiotherapy planning alone [8]. In the ORIOLE phase II trial, SABR improved outcomes and was enhanced by total consolidation of disease identified by PSMA-PET (baseline data blinded by protocol) [11]. However, data regarding the oncological outcomes (overall survival and progression-free after image-guided therapy) in those patients who underwent PSMA-PET during their diagnostic work-up are still missing, and the incidence of events (e.g., radiological and/or biochemical progression or deaths) needs to be further explored. Therefore, we aimed to evaluate the incidence of events during follow-up in hormone-sensitive prostate cancer (HSPC) patients who performed PSMA-PET in early stages of recurrence and were candidate to salvage therapy for BCR after radical therapy.

## Materials and methods

### Study design and participants

This a prospective analysis performed in a cohort of patients consecutively enrolled through a prospective single-arm study at our institution (Ethical Committee n. P-5315) [7]. All patients signed an informed consent form (ICF) before enrollment.

Inclusion criteria were (1) histologically proven PCa; (2) previous radical therapy, either radical prostatectomy (RP) or radiotherapy (RT); (3) proven BCR or biochemical persistence (BCP) after RP or RT, according to EAU guidelines [1]; (4) HSPC patients, androgen deprivation therapy-free (ADT-free) status for at least 6 months prior to PET scan; (5) at least 24 months of follow-up after PSMA-PET; (6) complete follow-up data available. Exclusion criteria were (1) patients not eligible for salvage therapy according to the uro-oncological tumor board; (2) castration resistant PCa (CRPC); (3) patients receiving androgen-receptor targeted therapy or chemotherapy; (4) inability to perform a PET scan.

All patients received PSMA-PET at a single referral center between November 2016 and September 2020. This study represents an update at mid-term follow-up of the single-arm prospective study about PSMA-PET diagnostic accuracy in HSPC patients, previously published by our group [7].

### Objectives

The primary objective was to evaluate the incidence of events occurred during follow-up in HSPC patients suitable for salvage therapy, who performed ^68^ Ga-PSMA-11 PET/CT during biochemical recurrence after radical treatment.

Secondary objectives were:

- To determine potential independent predictors of events;
- To evaluate the association between changes in clinical management occurred after PSMA-PET, assessed by a single-center multidisciplinary tumor board, and the incidence of events.

### Outcome measurements and statistical analysis

The primary outcome was the Event-Free Survival (EFS), defined as the time in months between the date of PSMA-PET examination and the date of event/last follow-up. Event was defined as one of the following conditions: (a) death; (b) radiological progression after PSMA-PET, defined as appearance of new PCa localization(s) at any imaging procedure performed during follow-up according to best standard of care (including bone scan, contrast-enhanced CT, whole-body MRI, PET/CT with PSMA or choline or fluciclovine); (c) PSA progression occurred after salvage radiotherapy in prostate bed and/or metastases directed therapy (e.g., SABR) and/or hormonal therapy alone or in association with radiotherapy.

Event-free patients were censored at the date of last follow-up assessment (study cut-off date October 1st 2020). The Kaplan–Meier method was used to estimate the EFS curves, comparing the effect of different predictors by the log-rank test. EFS was also investigated by the uni- and multivariate Cox proportional hazards regression, comparing the covariates by the Wald test and calculating 95% confidence interval (CI). The following variables were tested as potential predictors for EFS: age (> 70 vs ≤ 70 years), T (3a-4 vs 1–2), ISUP (3–5 vs 1–2), PSA, PSA doubling time (PSAdt), PET results (positive vs negative), change of management after PSMA-PET result (any vs none), salvage therapy (yes vs no), and clinical setting (BCP vs relapse after SRT vs first BCR). PSAdt was calculated according to Khan et al. [12], as previously reported [7]. For all survival estimations, PSA and PSAdt have been stratified using 0.5 ng/mL and 6 months as cut-off, respectively. The probability of a positive PSMA-PET result has been also estimated by a complete series of uni- and multivariate binary logistic regression models. While the dependent variable was the PSMA-PET result (positive vs negative), the potential determinants were pT stage, ISUP, PSA at the time of PET imaging, PSA doubling time, and clinical setting.

Changes in patient clinical management were defined by a single-center tumor board prior to PSMA-ET (considering clinical, pathological and laboratory data, performance status and PSMA-PET results) as previously reported [7] and showed in Supplementary Table 1. Three different clinical settings of PSA relapse prior to PSMA-PET were defined: first-time BCR (subgroup 1), defined as patients who achieved complete PSA response after primary therapy (surgery ± adjuvant RT, or primary RT) and subsequently experienced first BCR; patients who experienced PSA recurrence after prostate-bed SRT (subgroup 2); BCP after RP (subgroup 3), defined as PSA ≥ 0.1 ng/mL at 6 weeks after RP.

Patient characteristics at baseline were reported as absolute/relative frequencies for categorical variables and median (inter-quartile range [IQR]) for continuous ones. The Mann–Whitney and Kruskal–Wallis tests for continuous covariates, while the Fisher’s exact test for categorical ones were used for the inferential statistics, respectively. All reported *p* values were two-sided, at the conventional 5% significance level. Data were analyzed as of February 2021 using R 4.0.3 (R Foundation for Statistical Computing, Vienna-Austria, www.r-project.org).

### Procedures and image interpretation

^68^ Ga-PSMA-11 was synthesized in the radiochemistry laboratory of the Division of Nuclear Medicine of the AOU Città della Salute e della Scienza, University of Turin, as previously reported [7] and showed in Supplementary Table 1, in accordance with procedure guidelines [13, 14]. ^68^ Ga-PSMA-11 (1.8–2.2 MBq/kg) was injected intravenously. ^68^ Ga-PSMA-11 PET was performed in accordance with a standard technique, as previously reported [7]. All patients underwent PET/CT scan in a dedicated tomograph (Gemini Dual, Philips HealthCare). A low-dose CT scan was performed for attenuation correction of the PET emission data. In case of inconclusive findings at standard images, late pelvic scans were acquired at 120 (± 15) min post-injection, 6 min per bed position, 2 beds centered on pelvis. PET/CT images were locally analyzed with dedicated workstation (Advantage; GE Healthcare), and independently reviewed with consensus by two experienced nuclear medicine physicians. Images were interpreted in a per-region analysis, according to E-PSMA procedure guidelines [14][14]. Any focal tracer uptake higher than the surrounding background and not associated with physiological uptake was considered suspicious for malignant lesion. Definition of oligometastatic disease was defined by the presence of three or fewer PSMA positive metastases (M1a and/or M1b and/or M1c).

## Results

### Patients’ cohort

**Fig. 1:**
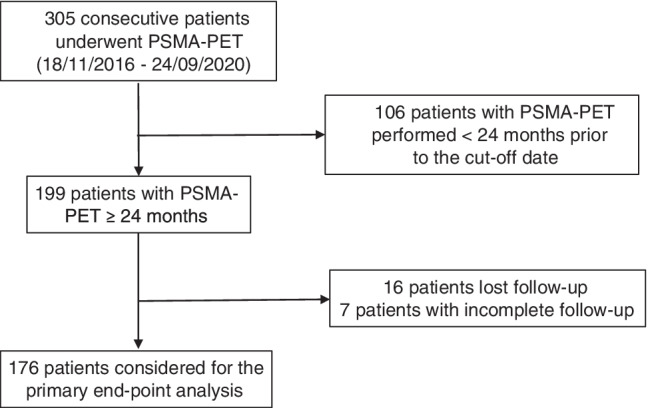
Study profile

Three hundred and five (*n* = 305) consecutive HSPC patients were prospectively enrolled and investigated with PSMA-PET at a single referral center (Nuclear Medicine, University Hospital of Turin) between November 2016 and September 2020. One hundred seventy-six (*n* = 176) patients matched the inclusion/exclusion criteria of this mid-term follow-up analysis and were considered eligible for the primary end-point analysis. Enrollment flow-chart is reported in detail in Fig. 1. Demographics and clinical characteristics of the study population are presented in Table 1. Median PSA at the time of PET was 0.62 (IQR 0.43–1.00) ng/mL while median PSAdt was 9.8 (IQR 4.7–18.4) months. Median time from PSA and PET date was 21 days (IQR 7–37 days).

**Table 1:** Population characteristics (*n* = 176)

Clinical features		Median	IQR
Age (years)		73	69–87
iPSA (ng/mL)		7.9	5.28–12.00
PSA at PET scan (ng/mL)		0.62	0.43–1.00
PSAdt at PET scan (months)		9.8	4.7–18.35
PSAvel at PET scan (ng/mL/year)		0.5	0.2–1.1
Clinical features		**Frequency % (*n*)**	
ISUP grade	1	14.8% (26)	
	2	25.6% (45)	
	3	27.8% (49)	
	4	15.3% (27)	
	5	11.9% (21)	
	Missing	4.5% (8)	
pT stage	< 3a	51.1% (90)	
	≥ 3a	44.9% (79)	
	Missing	4.0% (7)	
pN stage	N1	6.3% (11)	
R (margin)	R1	38.6% (68)	
Time to PSA relapse from primary therapy (months)	< 12	25.0% (44)	
	≥ 12	75.0% (132)	
Primary therapy	RP ± LND ± adjuvant RT	96.0% (169)	
	Primary RT	3.9% (7)	
Clinical setting of PSA failure	First BCR (subgroup 1)	43.2% (76)	
	PSA relapse after SRT in prostate bed (subgroup 2)	43.2% (76)	
	BCP after RP (subgroup 3)	13.6% (24)	

### PET results and clinical management

**Fig. 2:**
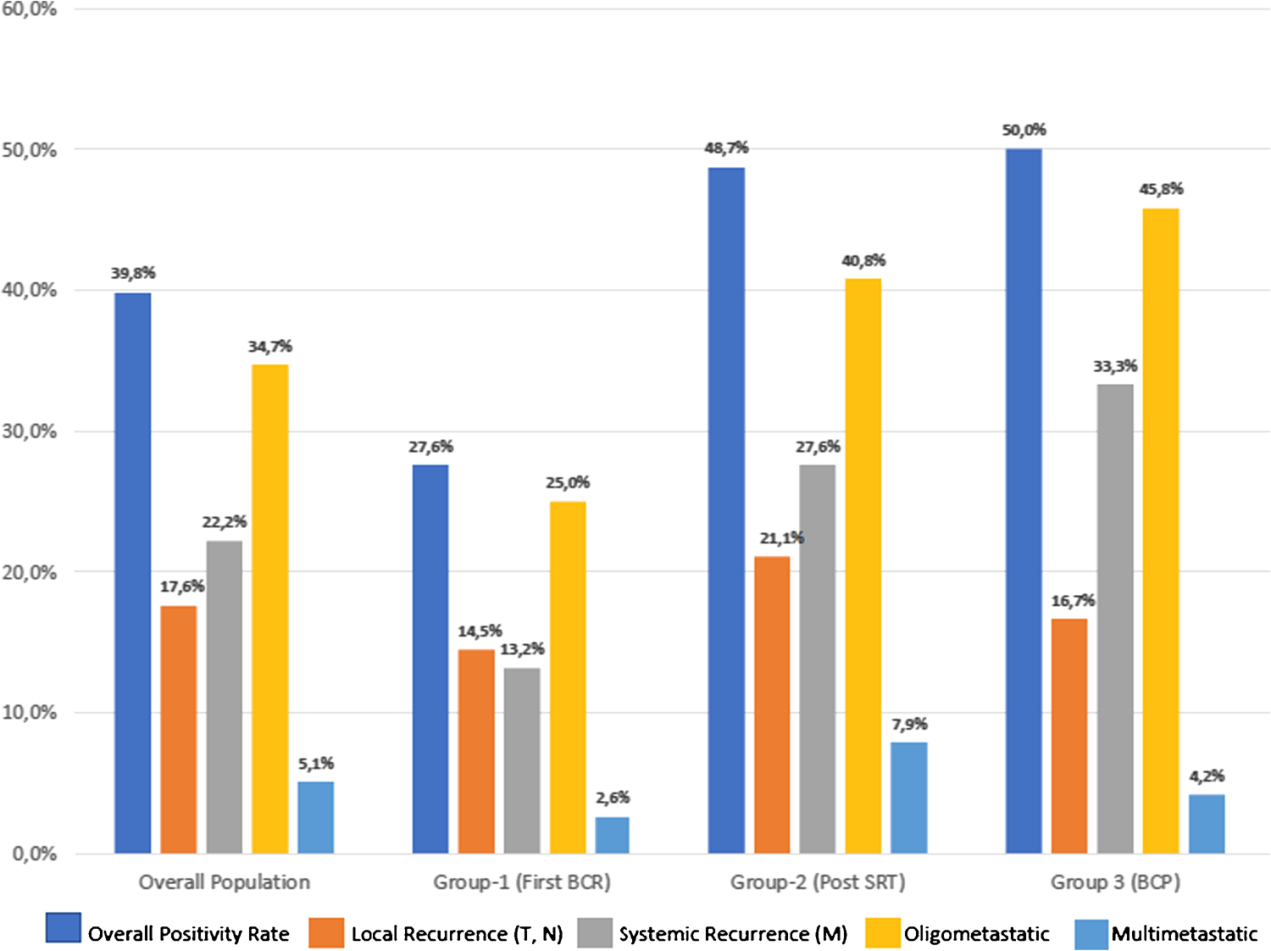
Rates of positivity for PSMA-PET in a population of biochemically recurrent HSPC patients, prospectively enrolled. The rates of positivity have been reported for pelvic vs systemic recurrence, and for oligo-metastatic (up to 3 lesions) vs multimetastatic disease. Data have been reported for the overall population and stratified by clinical setting

The 39.8% of the scans were interpreted as positive for PCa locations. All scans reported as positive reached a score for readers’ confidence of 4 or 5, as reported by the E-PSMA reporting system [14]. According to molecular imaging TNM (miTNM) definition [14, 15], prostate bed relapse (miTr) was detected in 6.3% of cases (11/176), pelvic nodes (miN1) in 18.8% (33/176), extra-pelvic nodes (miM1a) in 10.2% (18/176), bone metastasis (miM1b) in 12.5% (22/176), and visceral non-nodal metastasis (miM1c) in 2.8% (5/176). The overall presence of metastatic PSMA-avid lesions (miM1a, miM1b, miM1c) was observed in 22.2% of cases (39/176). Oligometastatic disease (1 to 3 PSMA positive lesions) was observed in 34.7% (61/176) of cases. Figure 2 and Table 2 summarize these results, stratified by clinical setting.

**Table 2:** Patients’ characteristics stratified by clinical setting

Clinical setting	PSA at PET ng/mL median (IQR)	ISUP ≥ 3% (*n*)	Positive PSMA-PET % (*n*)	Pelvic vs systemic recurrence	Oligo- vs multi metastatic
Overall (*n* = 176)	0.62 (0.43–1.00)	55.1% (97)	39.8% (70)	Pelvic: 17.6% (31) Systemic: 22.2% (39)	Oligo: 34.7% (61) Multi: 5.1% (9)
Subgroup 1 (*n* = 76)	0.58 (0.36–0.87)	48.7% (37)	27.6% (21)	Pelvic: 14.5% (11) Systemic: 13.2% (10)	Oligo: 25.0% (19) Multi: 2.6% (2)
Subgroup 2 (*n* = 76)	0.74 (0.5–1.25)	55.3% (42)	48.7% (37)	Pelvic: 21.1% (16) Systemic: 27.6% (21)	Oligo: 40.8% (31) Multi: 7.9% (6)
Subgroup 3 (*n* = 24)	0.45 (0.37–0.86)	75.0% (18)	50.0% (12)	Pelvic: 16.7% (4) Systemic: 33.3% (8)	Oligo: 45.8% (11) Multi: 4.2% (1)

*Subgroup 1* first BCR, *Subgroup 2 *PSA relapse after prostate-bed SRT, *Subgroup 3* BCP after RP

The following treatments were administrated after PSMA-PET: 52/176 clinical follow-up, 5/176 salvage pelvic lymph node dissection, 63/176 prostate bed salvage radiotherapy, 23/176 androgen deprivation therapy, 31/176 metastasis directed therapy, 1/176 salvage radical prostatectomy, 1/176 other. The multidisciplinary tumor-board performed changes to treatment planned prior to PSMA-PET scan in 30.1% of cases (53/176): 34.2% (26/76) of cases in subgroup 1, 19.7% (15/76) in subgroup 2 and 50% (12/24) in subgroup 3. Results concerning changes in treatment management are displayed in detail in Table 3.

**Table 3:** This table represents only the changes in therapeutic management occurred after PSMA-PET (stratified by clinical setting). Changes in the planned treatment prior to PSMA-PET were performed according a single-center multidisciplinary tumor board

Changes occurred in planned therapy management, after PSMA-PET	FUP	S-PLND	SRT	ADT	SABR (MDT)	S-RP
% (*n*)	% (*n*)	% (*n*)	%(*n*)	% (*n*)	% (*n*)	% (*n*)
Overall population 30.1% (53/176)	8.0% (14/176)	1.7% (3/176)	–	13.6% (24/176)	6.8% (12/176)	–
Subgroup 1 34.2% (26/76)	15.8% (12/76)	2.6% (2/76)	–	5.2% (4/76)	10.5% (8/76)	–
Subgroup 2 19.7% (15/76)	–	–	–	19.7% (15/76)	–	–
Subgroup 3 50.0% (12/24)	8.3% (2/24)	4.2% (1/24)	–	20.8% (5/24)	16.7% (4/24)	–

*FUP* Clinical follow-up with no therapies administered, *S-PLND* salvage pelvic lymph node dissection, *SRT* prostate-bed salvage radiotherapy, *ADT* androgen deprivation therapy, without other concomitant therapies, *SABR *stereotactic ablative radiotherapy, *MDT *metastasis directed therapy, *MDT* metastasis-directed therapy, *S-RP* salvage radical prostatectomy

In the multivariate logistic regression model, PSA > 0.5 ng/mL, PSAdt ≤ 6 months and pT stage ≥ 3a confirmed to be predictors of a positive PSMA-PET (p < 0.001), as previously reported [7].

### Survival analyses—primary objective

**Fig. 3:**
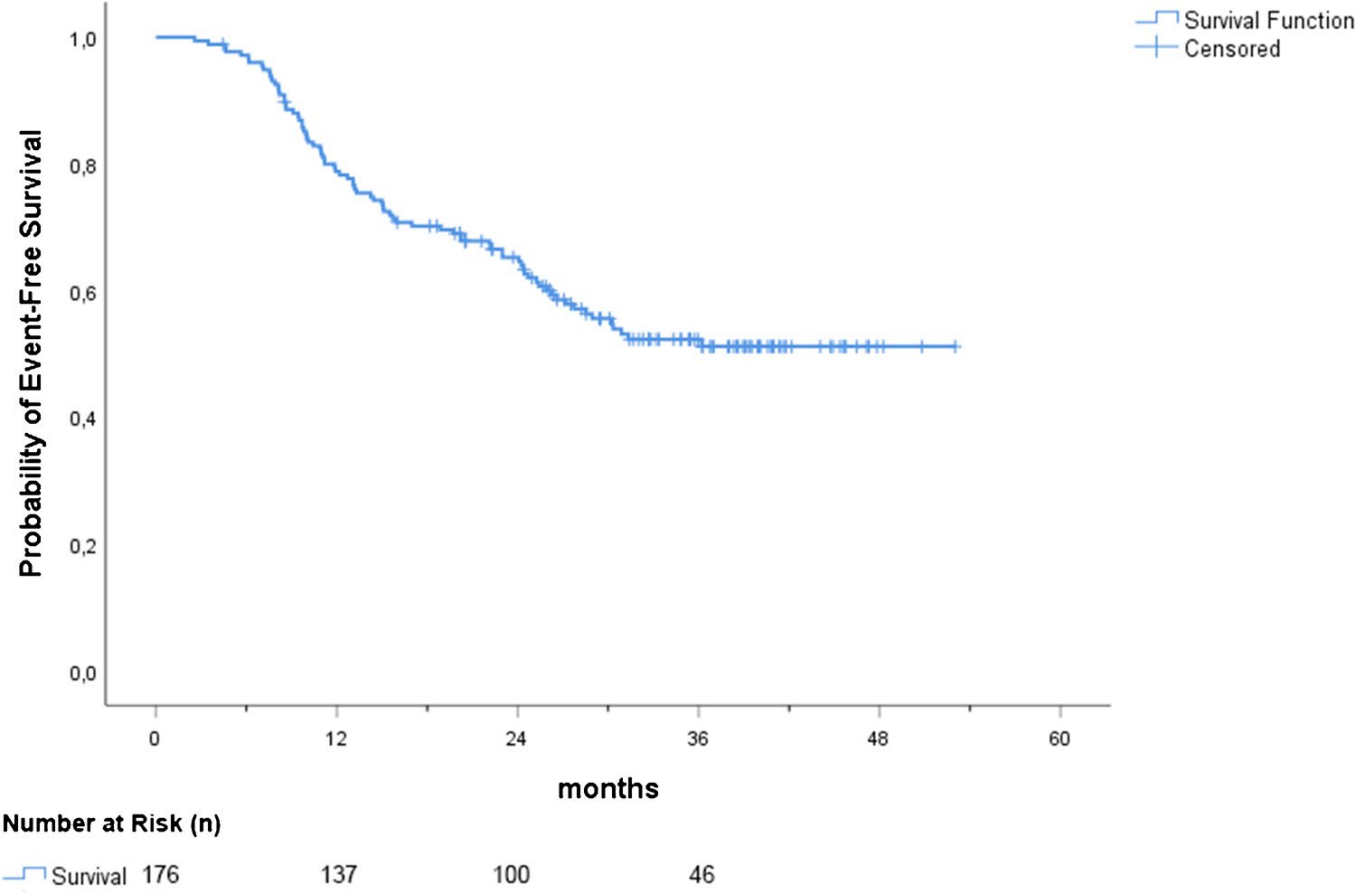
EFS for the overall population

The median follow-up for the whole cohort was 35.4 (IQR 26.5–40.3) months. Events were detected in 44.9% (79/176) of patients: 35.5% (27) in subgroup 1, 52.6% (40) in subgroup 2, and 50.0% (12) in subgroup 3. Event occurrences, stratified by clinical setting, are reported in detail in Table 4. While median EFS was not reached in the overall population, the median EFS in subgroups 2 and 3 were 25.4 and 30.9 months, respectively. In the overall population, the proportion of event-free patients was 78.8% at 1 year, 65.2% at 2 years, and 52.2%, at 3 years as shown in Fig. 3. The analysis of event-time distributions by the log-rank test proved that PSA value > 0.5 ng/mL (*p* = 0.003), PSAdt ≤ 6 months (*p* < 0.001) and a positive PSMA-PET scan (*p* < 0.001) were associated with a poorer EFS.

**Table 4:** Rate and type of events in the overall population and stratified by clinical setting

Clinical setting	Events	Type of Events		
		PSA recurrence after therapy*	Radiological progression**	Death
	% (*n*)	% (*n*)	% (*n*)	% (*n*)
Overall (*n* = 176)	44.9% (*n* = 79/176)	23.9% (*n* = 42/176)	17.6% (*n* = 31/176)	3.4% (*n* = 6/176)
Subgroup 1 (*n* = 76)	35.5% (*n* = 27/76)	25% (*n* = 19/76)	10.5% (*n* = 8/76)	0.0% (*n* = 0/76)
Subgroup 2 (*n* = 76)	52.6% (*n* = 40/76)	21.1% (*n* = 16/76)	26.3% (*n* = 20/76)	5.3% (*n* = 4/76)
Subgroup 3 (*n* = 24)	50.0% (*n* = 12/24)	29.2% (*n* = 7/24)	12.5% (*n* = 3/24)	8.3% (*n* = 2/24)

^*^Considering both radiotherapy (or S-PLND) and systemic therapy (ADT)

^**^Appearance of new PCa localization(s) at any imaging procedure including bone scan, CT, whole-body MRI, PET (PSMA or choline or fluciclovine)

### Survival analysis – secondary objectives

**Fig. 4:**
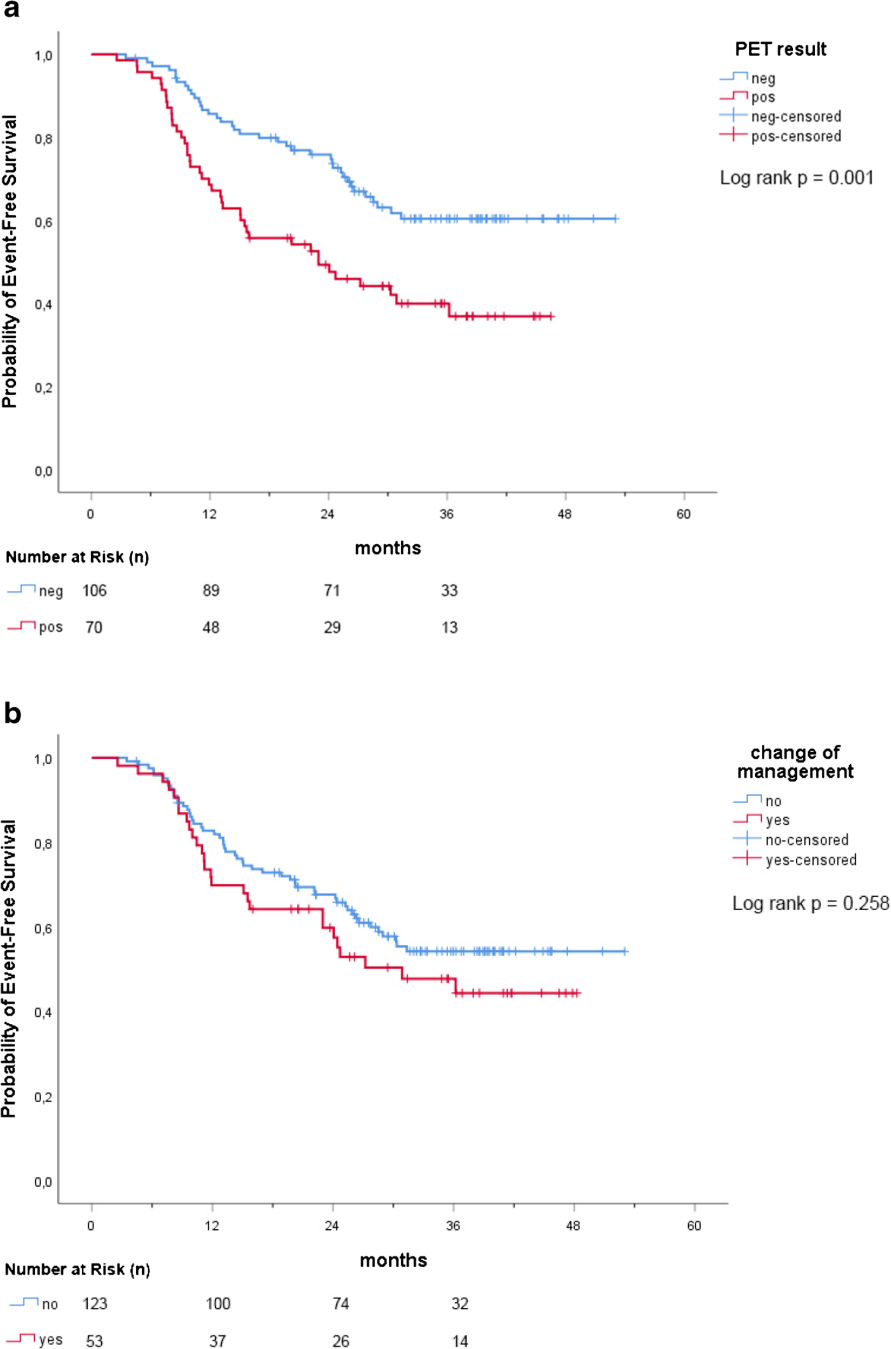

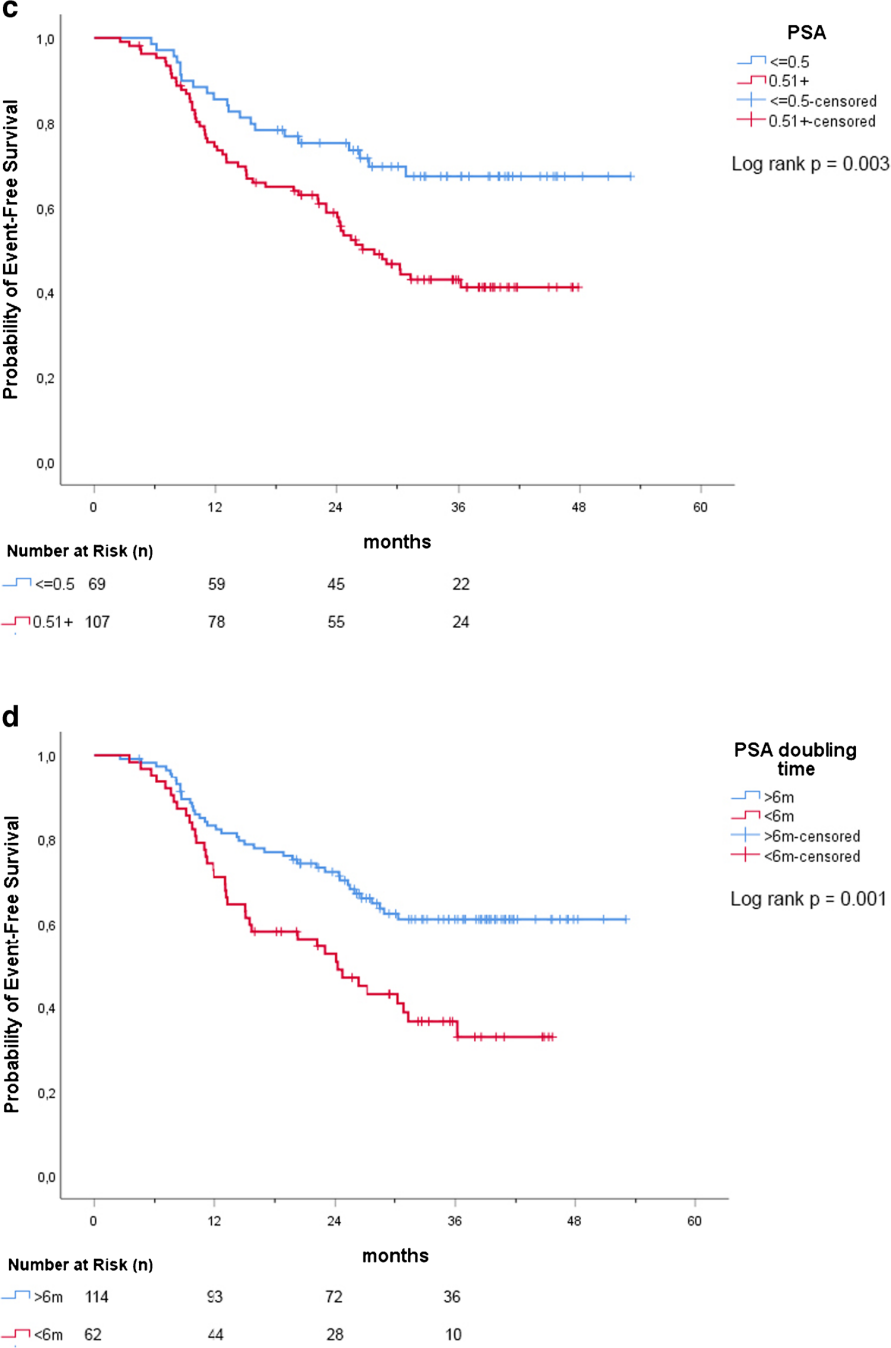
**a**–**d** EFS stratified by potential predictors PSA at PET scan, PSAdt, PET results, and change of management

In the overall population, no significant differences (*p* = 0.258) of event rates were observed stratifying the population by change of therapy management (change in therapy management after PSMA-PET vs. no change) (Fig. 4).

In the Cox univariate regression models, PSA > 0.5 ng/ml (HR 2.08; 95CI%: 1.26–3.42; *p* = 0.004), PSAdt ≤ 6 months (HR 2.03; 95CI%: 1.30–3.16; *p* = 0.002), positive PSMA-PET scan (HR 2.08; 95CI%: 1.34–3.24; *p* = 0.001) and clinical setting (subgroup 2 vs 1: HR 1.73; 95CI%: 1.06–2.82; *p* = 0.028) resulted significant event predictors. In the multivariate model, these associations were confirmed for PSA (HR 2.07; 95CI%: 1.25–3.41; *p* = 0.005) and PSAdt (HR 2.03; 95CI%: 1.29–3.17; *p* = 0.002), as reported in Table 5. No significant associations were observed regarding other variables.

**Table 5:** Univariate and multivariate Cox regression models for EFS

Potential EFS predictors	Univariate model			Multivariate model		
	HR	95% CI	*p*	HR	95% CI	*p*
Age (71 + vs ≤ 70 years)	0.93	0.59–1.47	0.761	–	–	–
T (3a-4 vs 1–2)	1.47	0.94–2.31	0.091	0.98	0.59–1.61	0.921
ISUP (3–5 vs 1–2)	1.10	0.69–1.74	0.694	–	–	–
PSA at PET scan (0.51 + vs ≤ 0.50 ng/ml)	2.08	1.26–3.42	0.004	2.07	1.25–3.41	0.005
PSA doubling time (6 + vs ≤ 6 months)	2.03	1.30–3.16	0.002	2.03	1.29–3.17	0.002
Salvage therapy (yes vs no)	0.95	0.58–155	0.831	–	–	–
Clinical setting			0.077			0.315
Relapse after SRT vs first BCR BCP vs first BCR	1.73 1.66	1.06–2.82 0.84–3.28	0.028 0.145	1.46 1.49	0.87–2.44 0.71–3.09	0.151 0.289
PET result (pos vs neg)	2.08	1.34–3.24	0.001	1.53	0.91–2.55	0.108
Change of management (yes vs no)	1.31	0.82–2.08	0.259	–	–	–

## Discussion

The cohort analyzed in our study included HSPC, hormone-free patients in the early stage of recurrence (median PSA 0.62 [IQR 0.43–1.00] ng/mL), thus representing a potential low tumor burden population suitable for salvage therapy. The overall PSMA-PET positivity rate for locating disease recurrence was 39.8%, with a detection of oligo-metastatic disease in 34.7% and extra-pelvic disease in 22.2% of patients. The overall positivity rate was slightly lower compared to that obtained in registry trials and studies enrolling larger cohorts [3, 4, 32]. However, the population was in the early stage of recurrence with low median PSA value, thus probably reflecting a population at lower-risk of positivity for PSMA-PET [28, 33]. In this clinical setting, current guidelines recognized the lack of sensitivity of conventional radiological imaging [1, 16] and PSMA-PET emerged as a new generation imaging procedure able to correctly localize PCa recurrence allowing for imaging-guided treatment [3–5, 17]. However, the oncological outcomes of patients who underwent PSMA-PET during recurrence have not been extensively explored.

Accordingly, our study was designed to evaluate the incidence of events at mid-term follow-up (median follow-up was 35.4 months). Events were detected in 44.9% (79/176) of the overall population, with a lower likelihood of events in patients with first-time BCR compared to other clinical settings. When tested for event predictors, our data showed a time-dependent association between PSMA-PET result and EFS: patients tested negative at PSMA-PET had significant lower incidence of events. This finding could be related to the presence of lower tumor-burden, that might be reflected by the presence of micro-metastatic disease not detectable by molecular imaging (negative PSMA-PET) and thus probably defining a population at lower-risk of developing clinically relevant events at mid-term follow-up. On the contrary, PSMA-PET positivity is more likely associated with higher levels of PSMA-expression by the neoplastic tissue, leading to higher disease aggressiveness and worse oncological outcomes [18]. These findings confirmed our hypothesis regarding the prognostic value of PSMA-PET in recurrent HSPC patients with low PSA values, based on previous literature evidence showing the role of choline-PET and PSMA-PET in predicting PCa patients’ survival [19–25].

Our analysis was also aimed to test other potential predictors of events in our cohort. As expected, high PSA at PET scan values and short PSAdt were associated with EFS during follow-up, both considering the Kaplan–Meier method and multivariate Cox regression model. These parameters are also already known as possible predictors of PSMA-PET positivity [26–28], as confirmed in our study. PSMA-PET did not prove statistical significance in the multivariate analysis (probably due to the confounding effect of PSA and PSAdt). However, the analysis of the EFS curves showed that patients with negative scan vs positive scan did not have an equal event-time distribution and the event rate were significantly different (lower event rates in case of negative scan; log rank *p* = 0.001). As a consequence, in case of patients who have a rising PSA after primary therapy and should be under observation due to low-risk characteristics (e.g., elderly, low PSA, long PSAdt, low ISUP) [29], the presence of negative PSMA-PET scan might strengthen this decision as the likelihood of relevant events during follow-up might be lower (except for those PCa with low PSMA expression). However, this consideration might be supported by dedicated studies considering patients with low-risk characteristics. Conversely, PSMA-PET imaging can detect and visualize recurrent lesions unlike PSA and PSAdt. This can significantly alter the management of patients, potentially improving clinical outcomes [9].

New generation imaging, including PSMA-PET, offer accurate disease staging and significantly influence the management of recurrent PCa, leading to imaging-guided approaches thanks to an improved target delineation. Our study also evaluated the impact of PSMA-PET on patient management. The uro-oncological tumor-board performed changes to treatments planned prior to PSMA-PET scan in 30.1% (53/176) of cases, similarly to previous studies [6, 30]. The higher rate of management changes in subgroup 3 (50% vs 34.2% in subgroup 1 vs 19.7% in subgroup 2) can be explained by the higher accuracy of PSMA-PET to visualize recurrent lesions in BCP group (residual disease after surgery). In our study, when the cohort of patients was stratified by change of therapy management, the two groups (patients with change after PSMA-PET vs. no-change) showed an equal event-time distribution, and the event rate did not significantly differ (Fig. 4). This finding might be explained by the high diagnostic accuracy of PSMA-PET and its role in influencing treatment strategies. Despite having a higher baseline risk of clinically relevant events, patients with positive PSMA-PET scans will more likely undergo changes in therapy management that should impact on the natural history of the disease. However, this study was not powered to evaluate the outcome of PMSA-guided salvage treatments. Data derived by randomized controlled phase III trials powered for efficacy [9] will clarify if PSMA-guided treatments performed in these patients will produce a clinical net benefit, improving the progression-free and cancer-specific survival.

### Limitation

This study is not exempt from limitations. First, median EFS was not reached in the overall population due to a lower event rate in subgroup 1 (35.5%). However, all patients performed PSMA-PET scan at least 24 months prior to the study cut-off date, the median follow-up was 35.4 (IQR 26.5–40.3) months and median EFS was reached in subgroups 2 and 3. Second, a formal sample size calculation was not performed. Nevertheless, our cohort of patients was consecutively enrolled through a prospective single-arm study at our institution and the lower sample size of subgroup 3 (*n* = 24) did not affect the assessment of the primary endpoint. Third, considering the study design (mid-term follow-up of an on-going prospective study), we considered patients who performed either surgery or radiotherapy as primary treatment, thus representing a real-world scenario. These two sub-cohorts have two different definitions of biochemical failure, and PSA absolute value holds different significance in these two different patterns of relapse. However, the number of patients who performed radiotherapy as up-front treatment was very limited (*n* = 7) representing the 3.9% of the overall population, and we investigated our cohort with other variables as to assess all potential predictors of EFS. A post-hoc analysis comprehending only patients who experienced relapse after surgery have been performed. The multivariable analysis resulted consistent to the results observed in the overall cohort, without any statistical significance. Fourth, validation of positive findings was not feasible in all cases due to ethical and practical reasons. Incidence of false positive findings in this cohort was previously published by our group [7] and registry studies demonstrated high positive predictive value for PSMA-PET [4, 31]. PSMA-PET interpretation by independent blinded readers (not involved in the study design or data acquisition) and an inter-reader agreement analysis would have been preferable. However, all scans were interpreted independently, and final diagnosis was reached by consensus, according to the most recent procedure guidelines [14]. Finally, previous studies [3, 4] have already established a high inter-reader reproducibility for ^68^ Ga-PSMA-11 PET.

## Conclusion

This study evaluated the incidence of events during follow-up in HSPC patients who performed PSMA-PET for disease recurrence after radical treatment. Low PSA and long PSAdt were significant predictors of EFS. Furthermore, a lower incidence of events was observed also in patients having negative PSMA-PET, since longer EFS was significantly more probable in case of a negative scan. These findings might be helpful in the decision-making process of recurrent PCa, leading to a cost-effective management of patients in early stages of disease recurrence.

## Supplementary Information

Below is the link to the electronic supplementary material.

Supplementary file1 (DOCX 16 KB)

## References

[1] Mottet N, van den Bergh RCN, Briers E, Cornford P, De Santis M, Fanti S, et al. European Association of Urology Guidelines. 2020 Edition. 2020;presented at the EAU Annual Congress Amsterdam 2020.

[2] Emmett L, Metser U, Bauman G, Hicks RJ, Weickhardt A, Davis ID (2019). Prospective, multisite, international comparison of 18F-fluoromethylcholine PET/CT, multiparametric MRI, and 68Ga-HBED-CC PSMA-11 PET/CT in men with high-risk features and biochemical failure after radical prostatectomy: clinical performance and patient outcomes. J Nucl Med.

[3] Calais J, Ceci F, Eiber M, Hope TA, Hofman MS, Rischpler C (2019). 18F-fluciclovine PET-CT and 68Ga-PSMA-11 PET-CT in patients with early biochemical recurrence after prostatectomy: a prospective, single-centre, single-arm, comparative imaging trial. Lancet Oncol.

[4] Fendler WP, Calais J, Eiber M, Flavell RR, Mishoe A, Feng FY (2019). Assessment of 68Ga-PSMA-11 PET accuracy in localizing recurrent prostate cancer: a prospective single-arm clinical trial. JAMA Oncol.

[5] Pienta KJ, Gorin MA, Rowe SP, Carroll PR, Pouliot F, Probst S, et al. A phase 2/3 prospective multicenter study of the diagnostic accuracy of prostate-specific membrane antigen PET/CT with 18F-DCFPyL in prostate cancer patients (OSPREY). J Urol 2021:101097JU0000000000001698. 10.1097/JU.0000000000001698. 10.1097/JU.0000000000001698 PMC8556578 33634707

[6] Calais J, Fendler WP, Eiber M, Gartmann J, Chu F-I, Nickols NG (2018). Impact of 68Ga-PSMA-11 PET/CT on the management of prostate cancer patients with biochemical recurrence. J Nucl Med.

[7] Deandreis D, Guarneri A, Ceci F, Lillaz B, Bartoncini S, Oderda M (2020). 68Ga-PSMA-11 PET/CT in recurrent hormone-sensitive prostate cancer (HSPC): a prospective single-centre study in patients eligible for salvage therapy. Eur J Nucl Med Mol Imaging.

[8] Valle L, Shabsovich D, de Meerleer G, Maurer T, Murphy DG, Nickols NG (2021). Use and impact of positron emission tomography/computed tomography prior to salvage radiation therapy in men with biochemical recurrence after radical prostatectomy: a scoping review. Eur Urol Oncol.

[9] Calais J, Armstrong WR, Kishan AU, Booker KM, Hope TA, Fendler WP (2021). Update from PSMA-SRT Trial NCT03582774: a randomized phase 3 imaging trial of prostate-specific membrane antigen positron emission tomography for salvage radiation therapy for prostate cancer recurrence powered for clinical outcome. Eur Urol Focus.

[10] Schiller K, Stöhrer L, Düsberg M, Borm K, Devecka M, Vogel MME (2021). PSMA-PET/CT-based lymph node atlas for prostate cancer patients recurring after primary treatment: clinical implications for salvage radiation therapy. Eur Urol Oncol.

[11] Phillips R, Shi WY, Deek M, Radwan N, Lim SJ, Antonarakis ES (2020). Outcomes of observation vs stereotactic ablative radiation for oligometastatic prostate cancer: the ORIOLE phase 2 randomized clinical trial. JAMA Oncol.

[12] Khan MA, Carter HB, Epstein JI, Miller MC, Landis P, Walsh PW (2003). Can prostate specific antigen derivatives and pathological parameters predict significant change in expectant management criteria for prostate cancer?. J Urol.

[13] Fendler WP, Eiber M, Beheshti M, Bomanji J, Ceci F, Cho S (2017). 68Ga-PSMA PET/CT: Joint EANM and SNMMI procedure guideline for prostate cancer imaging: version 1.0. Eur J Nucl Med Mol Imaging.

[14] Ceci F, Oprea-Lager DE, Emmett L, Adam JA, Bomanji J, Czernin J (2021). E-PSMA: the EANM standardized reporting guidelines v1.0 for PSMA-PET. Eur J Nucl Med Mol Imaging.

[15] Eiber M, Herrmann K, Calais J, Hadaschik B, Giesel FL, Hartenbach M (2018). Prostate cancer molecular imaging standardized evaluation (PROMISE): proposed miTNM Classification for the interpretation of PSMA-ligand PET/CT. J Nucl Med.

[16] Trabulsi EJ, Rumble RB, Jadvar H, Hope T, Pomper M, Turkbey B (2020). Optimum imaging strategies for advanced prostate cancer: ASCO Guideline. J Clin Oncol.

[17] Fendler WP, Ferdinandus J, Czernin J, Eiber M, Flavell RR, Behr SC (2020). Impact of 68Ga-PSMA-11 PET on the management of recurrent prostate cancer in a prospective single-arm clinical trial. J Nucl Med.

[18] Ferraro DA, Rüschoff JH, Muehlematter UJ, Kranzbühler B, Müller J, Messerli M (2020). Immunohistochemical PSMA expression patterns of primary prostate cancer tissue are associated with the detection rate of biochemical recurrence with 68Ga-PSMA-11-PET. Theranostics.

[19] Giovacchini G, Guglielmo P, Mapelli P, Incerti E, Gajate AMS, Giovannini E (2019). 11C-choline PET/CT predicts survival in prostate cancer patients with PSA < 1 NG/ml. Eur J Nucl Med Mol Imaging.

[20] Giovacchini G, Incerti E, Mapelli P, Kirienko M, Briganti A, Gandaglia G (2015). [^11^C]Choline PET/CT predicts survival in hormone-naive prostate cancer patients with biochemical failure after radical prostatectomy. Eur J Nucl Med Mol Imaging.

[21] Giovacchini G, Picchio M, Garcia-Parra R, Briganti A, Abdollah F, Gianolli L (2014). 11C-choline PET/CT predicts prostate cancer-specific survival in patients with biochemical failure during androgen-deprivation therapy. J Nucl Med.

[22] Kwee SA, Lim J, Watanabe A, Kromer-Baker K, Coel MN (2014). Prognosis related to metastatic burden measured by ^18^F-Fluorocholine PET/CT in castration-resistant prostate cancer. J Nucl Med.

[23] Caroli P, De Giorgi U, Scarpi E, Fantini L, Moretti A, Galassi R (2018). Prognostic value of 18F-choline PET/CT metabolic parameters in patients with metastatic castration-resistant prostate cancer treated with abiraterone or enzalutamide. Eur J Nucl Med Mol Imaging.

[24] Celli M, De Giorgi U, Caroli P, Di Iorio V, Fantini L, Rossetti V (2021). Clinical value of negative 68Ga-PSMA PET/CT in the management of biochemical recurrent prostate cancer patients. Eur J Nucl Med Mol Imaging.

[25] Emmett L, Tang R, Nandurkar R, Hruby G, Roach P, Watts JA (2020). 3-year freedom from progression after 68Ga-PSMA PET/CT-triaged management in men with biochemical recurrence after radical prostatectomy: results of a prospective multicenter trial. J Nucl Med.

[26] Pereira Mestre R, Treglia G, Ferrari M, Pascale M, Mazzara C, Azinwi NC (2019). Correlation between PSA kinetics and PSMA-PET in prostate cancer restaging: a meta-analysis. Eur J Clin Invest.

[27] Ceci F, Castellucci P, Graziani T, Farolfi A, Fonti C, Lodi F (2019). 68Ga-PSMA-11 PET/CT in recurrent prostate cancer: efficacy in different clinical stages of PSA failure after radical therapy. Eur J Nucl Med Mol Imaging.

[28] Ceci F, Bianchi L, Borghesi M, Polverari G, Farolfi A, Briganti A (2020). Prediction nomogram for 68Ga-PSMA-11 PET/CT in different clinical settings of PSA failure after radical treatment for prostate cancer. Eur J Nucl Med Mol Imaging.

[29] Zaorsky NG, Calais J, Fanti S, Tilki D, Dorff T, Spratt DE, Kishan AU (2021). Salvage therapy for prostate cancer after radical prostatectomy. Nat Rev Urol.

[30] Farolfi A, Ceci F, Castellucci P, Graziani T, Siepe G, Lambertini A (2019). 68Ga-PSMA-11 PET/CT in prostate cancer patients with biochemical recurrence after radical prostatectomy and PSA <0.5 ng/ml. Efficacy and impact on treatment strategy. Eur J Nucl Med Mol Imaging.

[31] Morris MJ, Rowe SP, Gorin MA, Saperstein L, Pouliot F, Josephson DY (2021). Diagnostic performance of 18F-DCFPyL-PET/CT in men with biochemically recurrent prostate cancer: results from the CONDOR phase 3, multicenter study. Clin Cancer Res.

[32] Afshar-Oromieh A, da Cunha ML, Wagner J, Haberkorn U, Debus N, Weber W (2021). Performance of [^68^Ga]Ga-PSMA-11 PET/CT in patients with recurrent prostate cancer after prostatectomy-a multi-centre evaluation of 2533 patients. Eur J Nucl Med Mol Imaging.

[33] Bianchi L, Castellucci P, Farolfi A, Droghetti M, Artigas C, Leite J, et al. Multicenter external validation of a nomogram for predicting positive prostate-specific membrane antigen/positron emission tomography scan in patients with prostate cancer recurrence. Eur Urol Oncol. 2021; 18:S2588–9311(21)00217–0. 10.1016/j.euo.2021.12.002. 10.1016/j.euo.2021.12.002 34933814

