# Supplementary material for: Event-free survival after 68 Ga-PSMA-11 PET/CT in recurrent hormone-sensitive prostate cancer (HSPC) patients eligible for salvage therapy
Source: Eur J Nucl Med Mol Imaging. 2022 Feb 26;49(9):3257–68. doi: 10.1007/s00259-022-05741-9 (PMC9250462; doi:10.1007/s00259-022-05741-9)
Supplement: Supplementary file 1 — Supplementary file1 (DOCX 16 KB) [file 259_2022_5741_MOESM1_ESM.docx]

**SUPPLEMENT**

**Table 1**. Change of management criteria.

| **Clinical Setting** | **Intended treatment** | **Change of management** |
| --- | --- | --- |
| Subgroup 1 (First BCR after RP) and Subgroup 3 (BCP after RP) | Prostate-bed SRT ± ADT | - Image-guided SABR or S-PLND for oligometastatic disease  - Administration of ADT (more than 6 months) in case of multimetastatic disease |
| Subgroup 2 (BCR after SRT) | - ADT/active surveillance in case of negative imaging | - SABR or S-PLND guided by PSMA-PET |

*BCR: biochemical recurrence, BCP: biochemical persistence, SRT: salvage radiotherapy, ADT: androgen deprivation therapy, SABR: stereotactic ablative radiotherapy, S-PLND: salvage pelvic lymph node dissection, PSMA-PET: prostate specific membrane antigen/positron emission tomography*

**^68^Ga-PSMA-11 synthesis procedure**

Gallium-68 was produced with ^68^Ge/^68^Ga generator (ITG Isotope Technologies Garching GmbH, Germany). ^68^Ga-PSMAHBEDCC (Glu-NH-CO-NH-Lys-(Ahx)-[[68Ga]Ga(N,N’-bis-[2-hydroxy-5-(carboxyethyl)benzyl]ethylenediamine-N,N’-diacetic-acid]) (^68^Ga-PSMA-11) was prepared in a similar procedure as described by Eder et al.[1], and transferred to cassette-based automated synthesis module (Module miniAllinOne, Trasis S.A, Belgium). The whole procedure was performed in accordance with the Good Manufacturing Practices (GMP). The system provides a final report, and the resulting solution (final product) is subjected to standard quality controls.

**References**

[1] Eder M, Neels O, Müller M, Bauder-Wüst U, Remde Y, Schäfer M, et al. Novel Preclinical and Radiopharmaceutical Aspects of [68Ga]Ga-PSMA-HBED-CC: A New PET Tracer for Imaging of Prostate Cancer. Pharmaceuticals (Basel) 2014;7:779–96. https://doi.org/10.3390/ph7070779.
